# Structural Connectivity Variances Underlie Functional and Behavioral Changes During Pain Relief Induced by Neuromodulation

**DOI:** 10.1038/srep41603

**Published:** 2017-02-02

**Authors:** Richard L. Lin, Gwenaëlle Douaud, Nicola Filippini, Thomas W. Okell, Charlotte J. Stagg, Irene Tracey

**Affiliations:** 1 Oxford Centre for Functional Magnetic Resonance Imaging of the Brain (FMRIB Centre), University of Oxford, Oxford OX1 3UH, United Kingdom; 2 Nuffield Division of Anaesthetics, Nuffield Department of Clinical Neurosciences, University of Oxford, Oxford OX1 3UH, United Kingdom; 3 Nuffield Department of Clinical Neurosciences, University of Oxford, Oxford OX1 3UH, United Kingdom; 4 Oxford Centre for Human Brain Activity, Department of Psychiatry, University of Oxford, Oxford OX1 3UH, United Kingdom

## Abstract

An increased understanding of the relationship between structural connections and functional and behavioral outcomes is an essential but under-explored topic in neuroscience. During transcranial direct current stimulation (tDCS)–induced analgesia, neuromodulation occurs through a top-down process that depends on inter-regional connections. To investigate whether variation in anatomical connectivity explains functional and behavorial outcomes during neuromodulation, we first combined tDCS and a tonic pain model with concurrent arterial spin labelling that measures cerebral perfusion related to ongoing neural activity. Left dorsolateral prefrontal cortex (L-DLPFC) tDCS induced an analgesic effect, which was explained by reduced perfusion to posterior insula and thalamus. Second, we used diffusion imaging to assess white matter structural integrity between L-DLPFC and thalamus, two key components of the neuromodulatory network. Fractional anisotropy of this tract correlated positively with functional and behavioral modulations. This suggests structural dependence by the neuromodulatory process to induce analgesia with potential relevance for patient stratification.

The inter-relationship between structural and functional connectivity and behavioral measures is an important but under-explored topic in neuroscience. In the motor system, the diffusion tensor imaging (DTI) metric of white matter integrity, fractional anisotropy (FA), has been combined with a transcranial magnetic stimulation (TMS)-based physiological index of functional connectivity, demonstrating a close relationship between the structural and functional connectivity of two brain regions during action selection^1^ and action reprogramming^2^. Structural integrity has even been shown to predict resting-state functional connectivity across almost one thousand cortical regions^3^, though the behavioral implications of this relationship have not been addressed. In addition, the co-variation between structural connectivity and behavioral measures has been explored^4^,^5^, but these studies did not consider possible changes in functional correlations. Here, we aim to study the influence of structural integrity on both functional connectivity and behavioral outcomes in the context of neuromodulation for pain relief.

The investigation of non-invasive neuromodulatory techniques such as transcranial direct current stimulation (tDCS) as potential therapeutic tools in the management of chronic pain has rapidly grown in recent years^6^,^7^. When anodal tDCS, which is facilitatory to the underlying cortex, is applied to the left dorsolateral prefrontal cortex (L-DLPFC), increased pain thresholds^8^ and decreased reports of emotional dimensions of pain^9^,^10^ are observed in healthy subjects. In addition, anodal tDCS to L-DLPFC has been reported to reduce pain ratings in chronic pain patients^11^,^12^,^13^. However, despite these promising behavioral results, the neural mechanisms that underpin the analgesic effects of tDCS are still largely unknown.

Studies of experimental pain have repeatedly observed the engagement of DLPFC in the presence of placebo analgesia^14^,^15^,^16^. A common hypothesis for this placebo-induced relief suggests that the L-DLPFC can “[keep] pain out of the mind” by modulating the strength of coupling between thalamus and midbrain^17^, thereby inducing analgesia via activation of the powerful descending pain modulatory system within the brainstem^18^,^19^. Linked through the fronto-subcortical circuit^20^, the L-DLPFC and thalamus are well-established regions within the pain modulatory network^21^. Indeed, DTI has demonstrated that the structural connection between DLPFC and periaqueductal gray (PAG), one key antinociceptive region, passes through the thalamus^22^.

We hypothesized that the joint involvement of L-DLPFC and thalamus, mediated through their interconnection, might underlie the behavioral efficacy of L-DLPFC tDCS; and, further, that the resulting functional and behavioral outcomes could be explained by the integrity of white matter connections between these regions. Individuals with stronger connections would be expected to gain greater functional and behavioral outcomes.

To address our hypothesis, we studied functional changes induced by neuromodulation with the relatively novel technique of whole brain arterial spin labelling (ASL). ASL uses arterial blood as the endogenous tracer to derive blood flow measurements^23^. As it provides a quantitative measure of blood flow, two different tonic states (e.g. no pain and tonic pain) can be directly compared and any changes in measured blood flow interpreted as a related change in tonic neural activity within that region^24^. Therefore, this method allows us to use a clinically meaningful ongoing pain state and yet identify the concomitant neural activity underpinning its neuromodulation, which is not possible using BOLD fMRI. The technique has been optimized and applied by our group in recent literature^25^,^26^.

To produce such a tonic pain experience, we applied a capsaicin-based ongoing pain model in healthy volunteers in lieu of patients to circumvent clinical and drug related confounds^27^. Cutaneous application of capsaicin induces primary and secondary hyperalgesia, as well as ongoing pain sensations when high concentrations of capsaicin cream are used^28^. As such, it is a valuable and ethical experimental model of symptoms described by chronic pain patients.

Subjects were screened for response to capsaicin, and responders selected to undergo two fMRI sessions with concurrent capsaicin application: one with anodal and one with sham tDCS (Fig. 1). This experimental design allowed us to compare behavioral and relative regional cerebral blood flow (rCBF) changes between active and placebo neuromodulatory conditions. Prior to the fMRI sessions, a separate DTI scan was also performed on a different day to assess the structural integrity of the L-DLPFC–thalamic tract, via quantification of FA^29^.

Correlation analyses were performed with probabilistic tractography^30^ as well as tract-based spatial statistics (TBSS)^31^ to test for correlations between individual FA values and rCBF changes, and FA and behavioral pain rating changes.

## Results

### Neuromodulation of pain intensity

We tested whether a significant analgesic effect was observed after applying 20 minutes of 1 mA anodal tDCS to L-DLPFC. Comparing pre- and post- stimulation pain intensity ratings for the anodal and sham tDCS sessions, we found a significant stimulation by time interaction (Repeated Measures ANOVA F_(1,17)_ = 4.460, p < 0.05), but no main effect of stimulation (F_(1,17)_ = 3.879, p = 0.065) or time (F_(1,17)_ = 2.646, p = 0.122). Post-hoc tests revealed a significant reduction in pain intensity immediately after anodal tDCS (0.78 ± 0.35 points; mean ± SEM) (t(17) = 2.219, p = 0.04) but not after sham tDCS (0.13 ± 0.29 points; mean ± SEM) (t(17) = 0.467, p = 0.65) (Fig. 2). There was no difference in pre-stimulation pain intensity ratings between the two sessions (t(17) = 0.482, p = 0.64). It is important to note that the ongoing pain ratings induced by the 1% capsaicin cream were stable throughout the period of stimulation, as confirmed in our study (Supplementary Fig. 1) and prior publications^32^,^33^. Further, the lack of a significant effect of sham stimulation on pain ratings pre- compared to post- stimulation supports this being a stable experience. Self-reported tDCS-induced sensations, recorded after each session, did not differ between the anodal and sham tDCS conditions (p > 0.05 for all surveyed sensations; Supplementary Fig. 2).

### Neuromodulation of functional activity during L-DLPFC tDCS

Using whole brain ASL fMRI, we sought to determine whether cerebral blood flow changes occurred in the regions implicated in pain processing during anodal tDCS. The contrast rCBF map of Sham_[Stimulation−Pre-stimulation]_ − Anodal_[Stimulation−Pre-stimulation]_ was computed to dissociate placebo and other nonspecific influences possibly arising from the neuromodulation (Fig. 3A, Supplementary Table 1). Significantly higher blood perfusion was observed during sham tDCS compared with anodal stimulation in the left posterior insula and left thalamus (FWE-corrected p < 0.01; Fig. 3A). Left thalamic rCBF at the dorsal nuclei was also significantly higher during sham tDCS compared to anodal tDCS (FWE-corrected p < 0.01; Fig. 3A), which suggests the top-down inhibitory influence of L-DLPFC. Conversely, left primary motor cortex (M1), another prominent excitatory target in the tDCS literature^13^,^34^,^35^, showed significantly lower rCBF (FWE-corrected p < 0.01) for the sham session compared to the anodal session (Fig. 3A).

Since activity in the posterior insula reflects the presence of ongoing pain^24^,^36^, we performed a post-hoc analysis to extract the perfusion time course of the activated voxels in the left posterior insula (Fig. 3B). Higher perfusion to left posterior insula was consistently observed during the stimulation period of sham tDCS compared with anodal tDCS. The finding suggests that the tDCS-associated anti-nociceptive effect was present throughout the stimulation period.

Previous studies have shown that anodal tDCS leads to an increase in perfusion under the stimulating electrode, here the L-DLPFC, in the absence of ongoing pain^26^,^37^. We therefore performed a ROI analysis to investigate blood perfusion changes in this region. Anodal tDCS was associated with an increased perfusion of 25.4 ± 6.9% and sham tDCS with −0.9 ± 1.9% perfusion change (average ± SEM) compared to the respective pre-stimulation period. The difference between these stimulation conditions was significant (t(17) = 2.116, p < 0.05). A significant increase in L-DLPFC rCBF (FWE-corrected p < 0.01) was also found during anodal stimulation (Anodal_[Stimulation−Pre-stimulation]_) but not during sham stimulation (Sham_[Stimulation−Pre-stimulation]_) (Supplementary Figs 3 and 4; Supplementary Tables 2 and 3).

### L-DLPFC–thalamic structural connectivity is related to pain intensity changes

We went on to investigate the relationship between the structural integrity of the white matter tract that we hypothesized was key to the behavioral effects of tDCS and the degree of analgesia induced by stimulation. The tracts between the L-DLPFC and left thalamus were identified using probabilistic tractography and were consistent across subjects (see Experimental Procedures for details) (Fig. 4A). Individual FA was then extracted from the reconstructed L-DLPFC-thalamic tracts for each subject. No outliers were detected in any of our datasets with Grubbs’ test (p < 0.01). We found a significant positive correlation across subjects (r = 0.654, p < 0.01) between individual FA and the rating difference during anodal tDCS sessions (Anodal_[Pre-stimulation−Stimulation]_), but not during sham tDCS sessions (Sham_[Pre-stimulation−Stimulation]_; r = 0.300, p = 0.23). Subjects who showed the greatest tDCS-induced decrease in pain ratings were those who had the greatest structural integrity in the L-DLPFC-thalamic tracts (Fig. 4B).

Some recently published studies have examined the effects of tDCS on brain chemistry and functional connectivity in pain patients and shown motor cortex connectivity to the thalamus (chemistry and connectivity to other structures) is specifically altered by tDCS^38^. Further, the baseline connectivity between these regions may predict successful tDCS analgesia in fibromyalgia^39^. As we observed tDCS effects on the M1 (Fig. 3), an analysis exploring whether there were relationships between the structural integrity of L-M1-DLPFC, L-M1-posterior insula, or L-M1-thalamus and tDCS induced analgesia was performed. We found no significant correlations (Supplementary Fig. 5).

Next, to test the specificity of the correlation between pain ratings and L-DLPFC-thalamic structural connectivity, we performed a voxelwise correlation using TBSS, an approach that explores the whole white matter without an *a priori* hypothesis. Using this approach, the only regions that showed a significant correlation between FA and tDCS-induced analgesia during anodal tDCS compared with during sham tDCS (Sham_[Stimulation−Pre-stimulation]_ − Anodal_[Stimulation−Pre-stimulation]_) were located near the anterior limb of the internal capsule, specifically within the L-DLPFC-thalamic connections previously identified using probabilistic tractography (FWE-corrected p < 0.05; Fig. 5). Notably, no voxels were found in the posterior limb of the internal capsule, which would have suggested left M1 involvement.

### L-DLPFC–thalamic structural connectivity is related to neuromodulation of functional connectivity

If the structural integrity of the connections between L-DLPFC and thalamus can affect the behavioral modulations by L-DLPFC tDCS, we anticipate that an effect should also appear in the functional connectivity between the two regions during ongoing pain as assessed using ASL. Specifically, we expected to observe an inter-dependence between individual FA within the L-DLPFC-thalamic tract and the functional connectivity (as measured by Pearson correlation coefficient) of L–DLPFC and left thalamus^3^. The correlation between tDCS-induced changes in this functional connectivity between the L-DLPFC and left thalamus and the individual L-DLPFC-thalamic tract mean FA was significant for anodal tDCS (r = 0.485, p < 0.05), but not for sham tDCS (r = −0.094, p = 0.71) (Fig. 6A). Subjects with the highest structural integrity between L-DLPFC and thalamus showed the highest functional coupling between these two structures during anodal tDCS. The contrast perfusion map between anodal and sham tDCS (Sham_[Stimulation−Pre-stimulation]_ − Anodal_[Stimulation−Pre-stimulation]_; Fig. 3A) also showed significant rCBF changes in thalamic voxels that overlapped with the voxels connected to the L-DLPFC as assessed using probabilistic tractography (Fig. 6B).

### L-DLPFC–thalamic structural connectivity is related to rCBF changes in posterior insula

Subjects with stronger L-DLPFC-thalamic connections showed higher analgesic response to L-DLPFC tDCS (Fig. 4B). This should be reflected in a greater change in their posterior insula activity between anodal and sham tDCS sessions given the region’s specific role in indicating ongoing pain^36^. We thus investigated the relationship between rCBF changes in the activated voxels of posterior insula (Sham_[Stimulation−Pre-stimulation]_ − Anodal_[Stimulation−Pre-stimulation]_; as shown in Fig. 3A) and individual L-DLPFC-thalamic tract mean FA (Fig. 7). This correlation was significant (r = 0.488, p = 0.04), further corroborating the correlation between subjects’ structural integrity and their behavioral response and suggesting the presence of functional modulation by anodal tDCS.

## Discussion

In summary, we demonstrated that functional and behavioral outcomes during neuromodulation to induce pain relief were related to structural integrity between key brain regions, in that stimulation of L-DLPFC modulates the thalamic activity downstream. The behavioral efficacy of tDCS applied to the L-DLPFC correlates with the structural integrity of the L-DLPFC–thalamic tract, which also positively relates to the functional connectivity between the two regions, as measured using ASL perfusion imaging – a new tool to explore the neural correlates of ongoing pain states.

Anodal tDCS produced a modest but significant reduction in the group pain intensity. The degree of this analgesic effect was significantly greater than that of sham tDCS, during which a smaller, non-significant decrease in pain intensity was observed. The magnitude of the analgesic effect induced by tDCS is consistent with previous findings^8^. Its high variability (0.78 ± 0.35 points on a 0–10 scale; mean ± SEM) between subjects is also common even among clinically approved analgesic treatments^34^,^40^ and directly supports our hypothesis that people may have different analgesic responses based upon their structural and functional circuitry.

tDCS-induced modulation of rCBF was measured with ASL in key pain-related regions throughout the brain. Recent task-free ASL-based studies during concurrent tDCS have strongly suggested that perfusion measures are not corrupted in the presence of ongoing tDCS^26^,^37^. Only regions within the stimulated hemisphere showed significant tDCS-induced modulation of rCBF. We observed a significant perfusion increase in L-DLPFC during anodal tDCS but not during sham stimulation, and a ROI analysis of the L-DLPFC region directly underneath the electrode found significantly higher rCBF under anodal compared to sham tDCS. Lack of wider functional changes throughout L-DLPFC may be due to possible placebo effects during the sham tDCS trials involving parts of the DLPFC^14^,^16^, and which might be differentially involved between the sham and anodal sessions. It is important to note that tDCS is not a focal technique, and it is unclear which sub-regions of the DLPFC are directly stimulated, though modelling studies suggest highest current densities directly under the stimulating electrode^41^,^42^.

The left M1 was more active during anodal tDCS than during sham tDCS. Increased perfusion to the ipsilateral motor cortex and increased functional connectivity between L-DLPFC and L-M1 have been shown during task-free anodal tDCS of L-DLPFC^26^. Strong projections from the prefrontal cortex, particularly the DLPFC, are known to reach the motor structures and exert behavioral control^43^. This pathway may explain our observation of left M1 perfusion changes. The L-M1 activity does not explain the subjects’ behavioral changes, as demonstrated by the lack of correlation between tDCS induced analgesia and the structural integrity between L-M1 and key pain-related regions (Supplementary Fig. 5). Furthermore, our TBSS analysis only demonstrated a significant relationship between structural connectivity and change in pain regions in the anterior thalamic radiation beyond the anterior limb of the internal capsule. M1, however, connects to the thalamus and other downstream pain-related structures via the pyramidal tracts through the posterior limb of the internal capsule^44^,^45^ and is hence unlikely to be involved in inducing analgesia for our subjects.

The contrast perfusion map helps to shed light on functional aspects of the pain neuromodulatory mechanism. L-DLPFC exerts top-down influences via the descending pain modulatory system^46^. The perfusion changes we observed in the thalamus support the hypothesis that the thalamus is involved in the pain modulatory process during L-DLPFC tDCS. The sham condition, which controlled for non-specific effects, was associated with an increased thalamic perfusion that was eliminated with the anodal condition, suggesting the presence of active modulation.

Robust structural connections link L-DLPFC and thalamus^20^, and the joint involvement of these two regions in the descending pain modulatory system has been described in past human studies^17^,^47^, as discussed in a recent review by Garcia-Larrea^19^. Studies with cognitive manipulation have also found increased DLPFC activity in the anticipation phase followed by reduced thalamic activation during pain stimulus. Indeed, the prefrontal cortex is at the ideal anatomical location to receive and modulate sensory and affective information^21^. DLPFC can inhibit medial and dorsal thalami to disrupt functional connection to the midbrain and induce pain relief^17^. This pathway is supported by animal literature^48^, where electrical stimulation of rat prefrontal cortex showed reduced midbrain activation to noxious stimuli on foot^49^. Similar stimulation of cat prefrontal cortex was associated with lower dorsal and medial thalamic activity during pain relief^50^. These studies strongly support to the presence of a descending pain modulatory network, upon which we used tDCS to provide pain relief.

As the main relay center between brain and body, the thalamus is well positioned to modulate nociceptive transmission from lower structures. This area is mainly responsible for somatosensory relay and motor function and dampening. White matter connections between the PAG and L-DLPFC pass through the thalamus^22^. An ongoing pain model with capsaicin has been shown to enhance perfusion of both lateral and medial dorsal thalami in the absence of any modulation^51^,^52^. These regions of the thalamus are associated primarily with the prefrontal cortex and cognitive functions and also correspond to the voxels where we observed higher rCBF in the sham condition during ongoing pain compared to anodal condition. Structural and functional studies have both shown these areas to connect downstream to PAG^17^,^22^,^47^,^53^. Therefore, the analgesic effects of L-DLPFC anodal tDCS may occur through thalamic downregulation via this network.

Furthermore, the left posterior insula activity, which was contralateral to the capsaicin site on the right calf, had a significantly higher rCBF throughout sham tDCS than anodal tDCS (Fig. 3). The contralateral posterior insula reflects the presence of nociceptive processing in the dorsal horn from the ongoing pain^24^ and commonly increases in acute pain studies^54^ as well as showing nociceptive somatotopic organization^55^. This concept was reaffirmed in a recent study by our group using ASL to explore the neural basis of ongoing pain^36^. The changes we observed in its activity corroborate the volunteers’ tDCS-induced analgesia. The tonic, top-down suppression of left posterior insula activity supports our hypothesis that anti-nociceptive effect is associated with active L-DLPFC stimulation.

The inclusion of diffusion imaging allowed us to examine the underlying structural effects of inter-regional connections on functional and behavioral modulation. Analysis with probabilistic tractography was used to identify the L-DLPFC–thalamic tract. The FA in this pathway significantly correlated with pain intensity changes during anodal tDCS and not during sham tDCS. TBSS analysis, which does not rely on any *a priori* hypothesis, separately verified this conclusion. Indeed, on the whole brain FA skeleton, the only white matter voxels that covaried with the tDCS-induced pain intensity changes were found within the L-DLPFC-thalamic pathway identified using tractography. No voxels outside of this pathway showed a significant correlation, which suggested that the relationship was specific to the L-DLPFC–thalamic tract. The significant relationship between individual L-DLPFC-thalamic FA and changes in posterior insula rCBF further corroborates the above results to suggest that the behavioral changes were secondary to neuromodulation through this pathway.

Comparing functional and structural measures, we found a significant covariance between the functional connectivity of L-DLPFC and thalamic activity during anodal tDCS and the FA values of their inter-connection. Structural connectivity has previously been shown to predict functional connectivity^3^, lending support to our hypothesis of top-down neuromodulatory influence by L-DLPFC tDCS. Inter-subject variation in myelination or axon density or diameter, which would alter respective FA values^56^, can contribute to individual differences in functional correlation. This could provide some participants with faster or better-coordinated axon conduction to affect the extent of neuromodulation^1^. The magnitude of the stimulation-induced modulation of functional connectivity therefore likely depended on the intrinsic strength of the structural connections.

The diffusion imaging results suggest that structural integrity between L-DLPFC and thalamus is an important factor in pain neuromodulation. A wealth of literature supports the importance of DLPFC, thalamus, and their connection, during pain relief. L-DLPFC has been shown to modulate pain sensation in placebo analgesia studies^14^,^15^,^57^,^58^, possibly by changing thalamic coupling to midbrain^17^. L-DLPFC is also highly involved in a person’s cognitive functions^58^, which raises the possibility of using anodal tDCS coupled to a cognitive behavioral treatment (CBT) for pain. In our study, the perfusion of L-DLPFC, a region also involved in attention^47^ and anticipation^58^, was altered during anodal tDCS. This presents a promising first step towards the establishment of coupling tDCS with pain-related CBT to produce possibly synergistic effects and greater outcomes, although further work is needed.

While our ASL approach has been applied and optimized previously^25^,^26^, we are limited by the current advancement of ASL that technically prevents us from performing region parcellation or exploring the brainstem without compromising whole brain exploration. By gaining the ability to measure the tonic effect of an ongoing pain paradigm superimposed by continuous tDCS, ASL relies on a lower resolution (4 × 4 × 4.6 mm^3^ voxels) to yield sufficient SNR for functional studies. Due to the relatively poor spatial resolution of ASL, we are unable to perform a parcellation of the thalamus or posterior insula and therefore are unable to comment precisely on which sub-regions of these structures show tDCS-related changes in activity. When technology allows, future ASL based research studying the modulatory mechanism of tDCS should investigate the involvement of brainstem, namely PAG, or the sub-regions of thalamus or posterior insula. These restraints, however, do not undermine our conclusions. For example, past literature has established the roles of thalamus and DLPFC in pain modulation without simultaneous investigation of brainstem functions^14^,^50^.

We used capsaicin as a model for some features of on-going clinical pain and explored how variances in FA impact its effectiveness. It is important to highlight that the effects of tDCS on neural function seen in this ‘model’ may not be exactly replicated in clinical populations. However, there is evidence from other clinical situations that the neural effects of tDCS are similar in healthy controls and in patients^59^,^60^ and that the neural effects of a single stimulation session are similar to those seen after multiple interventions^59^,^61^. Despite these results, it is the case that the long-term effects of multiple tDCS sessions in clinical populations should be directly studied. In particular, our main observation that the effectiveness of tDCS is related to the structural integrity of white matter tracts supports a personalized use of the intervention that takes into account possible structural variances between patients.

The current findings demonstrate a functional and behavioral dependence on the structural measures of the relevant inter-connections during pain neuromodulation. This relationship may explain some variations in the clinical efficacy of tDCS as a pain treatment tool^8^,^13^ and could be used as a predictor of patient response to the stimulation. We also believe that the methods developed here provide an overall framework to gain mechanistic insight into the effect of tDCS. This could widely benefit the study of other neuromodulatory applications, such as in emotion regulation and memory processing that may be influenced via structural integrity in a similar manner.

## Methods

### Subject Recruitment

All experimental protocols were approved by Oxfordshire Research Ethics Committee C. In accordance with ethical regulations from the U.K. National Research Ethics Service and the Declaration of Helsinki (1996), all research participants were fully informed of the techniques used and gave written consent to participate in the study. Fifty-one healthy subjects (right handed; age: 18–45 years; 23 females) were initially screened for their capsaicin response. None of these volunteers were taking any prescription medications, antidepressants, or pain medications throughout their study participation, and all were fully informed of the techniques involved and the potential risks associated with tDCS^62^, MRI, and capsaicin and gave their informed consent to participate, in accordance with local ethics committee approval. However, they were not aware of the specific aims of the study. From this group, nineteen capsaicin responders participated in the remainder of the study. One volunteer eventually withdrew due to sensations of discomfort during the first fMRI session, which involved sham tDCS, and was hence excluded in the data analysis. The final subject cohort (N = 18; 6 females) had a mean age of 24.5 years with a standard deviation of 5.4 years and was recruited from the Oxford region.

### Tonic Pain Model

Volunteers received a topical patch of capsaicin cream (1% w/w, Pharmasol Limited, Andover, UK) on the center of the medial side of their right calf. The volume used ranged between 5 ml and 10 ml (Density: 0.5 ml/cm^2^) depending on the subject’s response. If a subject underwent multiple sessions of capsaicin screening to calibrate for the appropriate capsaicin volume, a minimum gap of one week was maintained between sessions to allow neuronal recovery and prevent tachyphylaxis after capsaicin desensitization^63^.

To record the ongoing pain intensity ratings, we used a continuous 0–10 rating scale that was anchored between “None” and “Worst Imaginable.” The scale was programmed with Presentation software (Version 14.5, Neurobehavioral Systems, Albany, CA, USA) and was placed beside the subject on a notebook computer. The participants were instructed to give a rating whenever they felt a change in pain, and they had full control of the scale throughout the experiment by moving it with the right or left arrow key. This setup also minimized the subjects’ interactions with the experimenter and thus reduced any possible interference factors. Unknown to the subjects, their ratings were monitored on a separate screen to track their progress. Each experiment session lasted from 1 to 2 hours depending on the individual response. Subjects were considered to be capsaicin responders if they maintained a consistent pain intensity rating between 5 and 7 (on a scale from 0 to 10) for at least 30 minutes.

### Study Setup and Design

Participants received sessions of anodal and sham tDCS that were counterbalanced across the group. A gap of at least one week was spaced for every subject between the two experimental sessions. An MR-compatible tDCS kit (DC-Stimulator MR, Magstim, Cardiff, UK) was used, which was fitted with a pair of 5 kΩ resistors by the electrodes (7 cm × 5 cm) to prevent the occurrence of eddy currents or significant electrode heating in an MR setting. The active electrode was centered at the F3 position in EEG 10/20 system, the location for L-DLPFC used in previous tDCS-pain studies^8^,^13^. An Omega-3 oil pill (Ultra-Pure Omega-3 Fish Oil, Purity Products, Plainview, NY, USA) was placed above the electrode center in order to visualize the electrode’s location in MR images. The reference electrode was located above the contralateral supraorbital ridge. To minimize the contact resistance between the scalp and the electrodes, high-chloride EEG gel (Abralyt HiCl, EasyCap, Herrsching, Germany) was used as a conducting medium. As is standard, prior to each session of tDCS, participants were given approximately 15 seconds of stimulation in order to familiarize themselves with the sensation outside the scanner. tDCS of such short duration has been shown to exert no lasting after-effects^62^.

**Figure 1:**
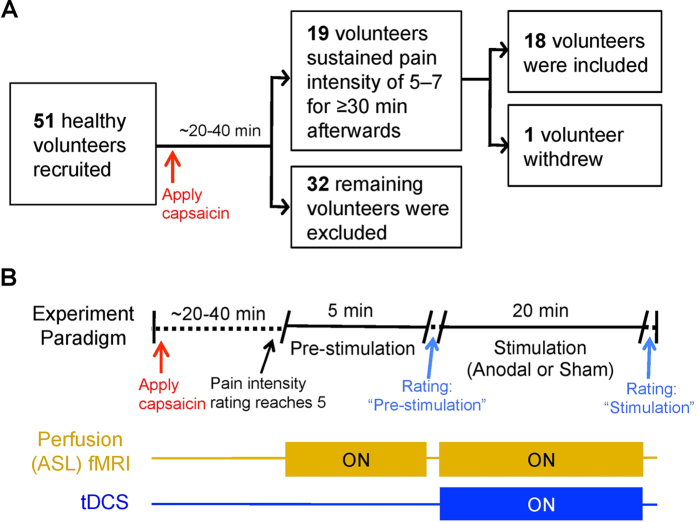
Experiment paradigm. (**A**) Fifty-one healthy volunteers were first screened for their response to topical application of 1% capsaicin cream on the right, medial calf. To achieve optimal temporal resolution, a continuous rating scale, anchored between 0 (none) and 10 (worst imaginable) was used to record the subject’s ongoing pain intensity. After capsaicin application for 20–40 minutes, only subjects who reached and maintained a tonic pain experience (pain intensity rating of 5–7 for ≥30 minutes; Supplementary Fig. 1) participated in the remainder of the study. One subject withdrew due to discomfort during a sham tDCS MRI session. (**B**) Eighteen capsaicin responders participated in two perfusion MRI sessions, one with anodal tDCS and one with sham tDCS, at least one week apart. The order of the experimental sessions was randomized and counterbalanced across the subject cohort. Capsaicin cream was applied as in the initial screening session. No subjects reported pain before the session commenced. Once the subject’s pain intensity rating reached a score of 5, which typically required 20–40 minutes, a 5-minute pre-stimulation ASL fMRI scan was performed followed by a 20-minute scan with concurrent tDCS (anodal or sham). Subjects’ pain ratings were verbally recorded after each scan using the same numerical rating scale, as above. Prior to ASL fMRI scans, subjects also underwent a DTI scan in a previous session on a different day.

**Figure 2:**
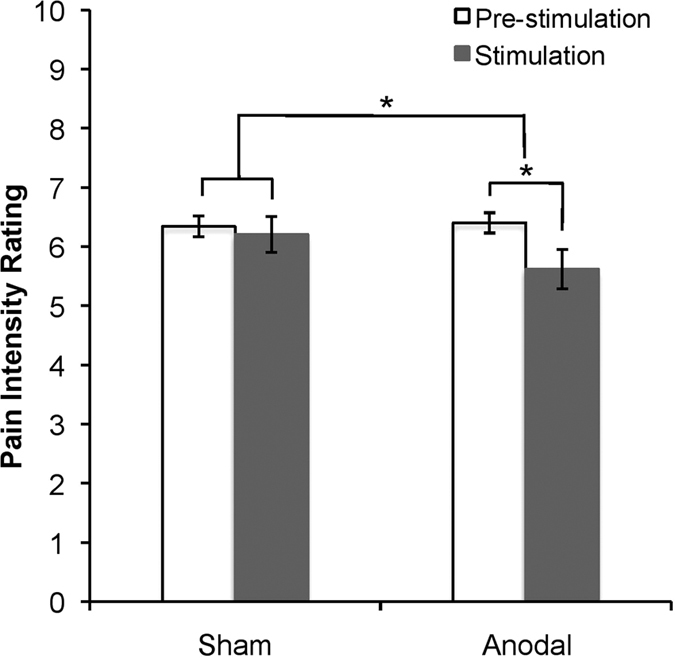
Behavioral pain response to L-DLPFC tDCS. Pain intensity ratings were recorded immediately before and after sham and anodal tDCS. The numerical rating scale was anchored between the extremes of 0 (“None”) and 10 (“Worst Imaginable”). Greater analgesia was found with anodal tDCS compared to sham tDCS (ANOVA stimulation by time interaction effect F_(1,17)_ = 4.460, p < 0.05). A significant reduction was also observed in anodal tDCS trials (p = 0.04) but not in sham tDCS trials (p = 0.65). (*indicates p < 0.05).

The experimental paradigm for the fMRI sessions is illustrated in Fig. 1B. Subjects experienced no pain before we placed topical capsaicin cream on their right calf in the scanner. The volumes and areas applied were identical to those during the initial screen for each subject. Subjects were instructed to alert the researcher when their pain intensity rating reached 5 on a 0–10 numerical rating scale (NRS), anchored on the extremes of “None” and “Worst Imaginable.” Once this rating was reached, we initiated a 5-minute pre-stimulation perfusion fMRI scan without tDCS. A concurrent tDCS-fMRI was then performed for 20 minutes between two 10-second ramp-up and -down periods. To increase the likelihood of detecting adverse events, the first 15 seconds of the stimulation were used to ensure that the subjects were comfortable before scanning began. For anodal tDCS sessions, subjects were given 1 mA current throughout the entire 20 minutes. For sham tDCS sessions, subjects experienced 30 seconds of stimulation before the tDCS kit was turned off. The subjects were blinded to the polarity of their stimulation sessions, and there were no significant differences in the reported sensations of anodal and sham tDCS^64^.

Before and after each scan, subjects verbally reported their numerical pain intensity ratings on the same NRS as above. When scoring, they were instructed to give any number between 0 and 10. Besides experiencing pain and providing their ratings, the subjects did not carry out any other tasks during the sessions and were asked to remain motionless and awake.

After each session, the subjects were asked to fill out a questionnaire regarding possible tDCS sensations, which consisted of tingling, itching, burning, tiredness, nervousness, concentration, headache, and visual problems^65^. If a question was answered “yes”, the subjects were then asked to rate the sensation intensity from 1 to 5 (1 = mild, 2 = moderate, 3 = average, 4 = severe, and 5 = intolerable). In addition, they were asked whether they saw a flash from tDCS due to retinal stimulation from the reference electrode. These ratings were then compared between anodal and sham tDCS sessions with a paired t-test, which found no significant differences in stimulation sensations (Supplementary Fig. 2).

Lastly, participants underwent a diffusion-weighted imaging scan on a previous experimental session on a different day, which allowed us to examine the structural integrity of their white matter.

### MRI Sequences

#### Structural Imaging

For registration purposes, the subjects underwent two repetitions of high-resolution (1 × 1 × 1 mm^3^ voxels) T1-weighted structural scans (to increase signal-to-noise ratio) on a 3 T MRI scanner (Magnetom Verio 3 T, Siemens Healthcare, Erlangen, Germany) using a 32-channel head coil (TR = 2.04 s, TE = 4.7 ms, FOV = 192 mm, and acquisition time = 6 min/scan).

#### Arterial Spin Labelling

Perfusion imaging was acquired with a pseudo-continuous ASL sequence that applied a gradient-echo EPI readout (TR = 3.48 s, TE = 13 ms, 6/8 k-space). This ASL portion of this sequence is similar to the non-selective cycles described in Okell *et al*.^23^ and has been previously optimized^25^ and applied^26^. Whole brain coverage was provided for each subject with 28 axial slices, which were acquired in ascending order (4 × 4 × 4.6 mm^3^ voxels) with an inter-slice gap of 0.46 mm. Using a time-of-flight scan of the neck, the optimal labelling plane was chosen at approximately 8 to 10 cm inferior to the center of the axial slices. The tagging pulse train lasted 1.4 s and was followed by a post-labelling delay of 900 ms. The high-resolution (1 × 1 × 1 mm^3^ voxels) structural scans were acquired with a standard T1-weighted sequence (TR = 2.04 s, TE = 4.68 ms) and contained 192 axial slices.

#### Diffusion Weighted Imaging

Diffusion scans were collected with a 3 T MRI scanner (3 T Trio, Siemens Healthcare, Erlangen, Germany) using a 12-channel head coil. An EPI diffusion-weighted (2 × 2 × 2 mm^3^ voxels) sequence (TR = 9.30 s, TE = 94 ms, FOV = 192 mm, and acquisition time = 20 min/scan) was applied in 60 isotropically-distributed gradient directions with a *b*-value of 1000 s/mm^2^. Each scan contained two repetitions to allow averaging during image analysis, which improves the overall signal-to-noise ratio.

### Data Analysis – ASL

#### Perfusion Measures

Separate first-level analysis of each scan was carried out with FMRIB software library version 4.1.7 (http://www.fmrib.ox.ac.uk/fsl)^66^. The functional data first underwent motion correction with a six-parameter affine algorithm. Automatic brain extraction was followed by manual correction to ensure thoroughness and precision. As previously^26^, we used non-linear high-pass temporal filtering (sigma of 200 s) to remove scanner drifts and BOLD contaminations and applied spatial smoothing (full-width half-maximum of 8 mm). The perfusion subtraction of the ASL data was modeled with a boxcar regressor by using the TR (3.48 s) as the duration of each on or off period. Finally, functional data was registered using an affine transformation to the respective high-resolution T1 structural scan, which was then non-linearly registered to Montreal Neurological Institute (MNI) standard brain.

**Figure 3:**
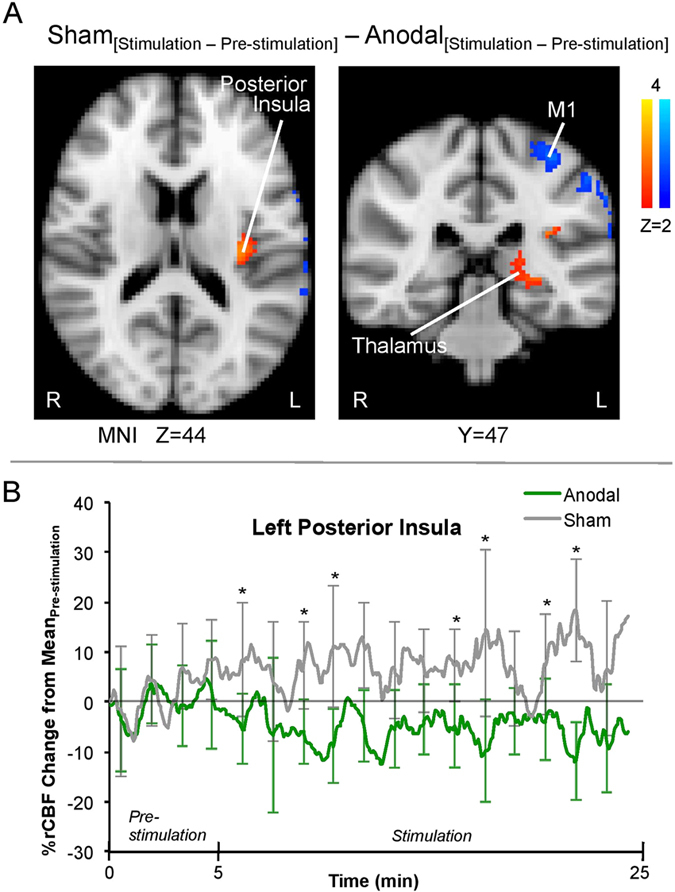
Functional modulation by L-DLPFC tDCS (FWE-corrected p < 0.01). (**A**) Perfusion activity map of gray matter associated with Sham_[Stimulation−Pre-stimulation]_ − Anodal_[Stimulation−Pre-stimulation]_ (mixed effects; Z > 2). The sham session showed higher (in yellow-red) rCBF to left posterior insula and left thalamus and lower (in blue) rCBF to the left M1 than the anodal session. The striking lateralization of significant rCBF changes suggests that the modulations originated from anodal tDCS of L-DLPFC. (**B**) Due to the positive relationship between ongoing pain and posterior insula activity, the perfusion time course of the activated voxels in the left posterior insula was extracted for illustrative purposes. Despite temporal variations across the periods, the rCBF of posterior insula was consistently higher for the sham session than for the anodal session after their respective pre-stimulation period. This finding supports the behavioral pain ratings reported by the research volunteers. All perfusion percentages were compared to the mean time course value of the pre-stimulation period in the respective stimulation session. The data points were smoothed with a moving average filter (period = 1 minute). (*indicates p < 0.05).

**Figure 4:**
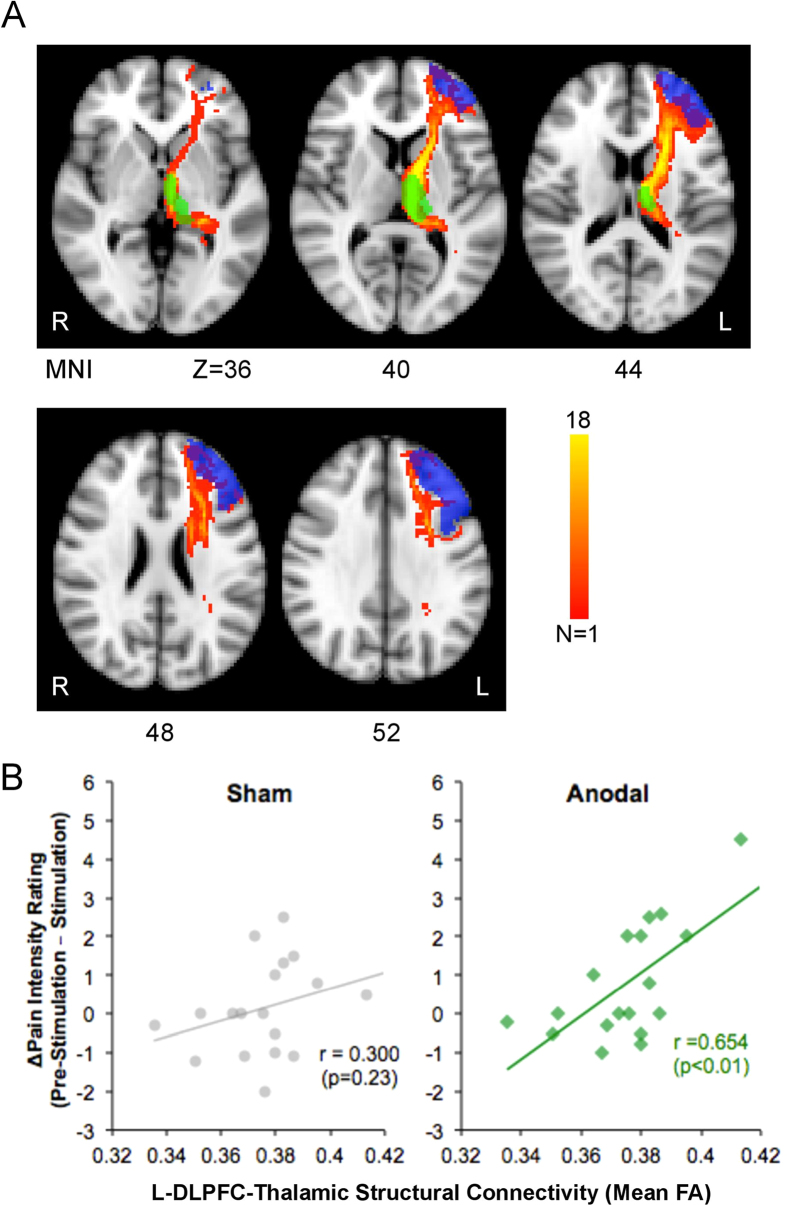
Structural connectivity between L-DLPFC and left thalamus correlates with analgesic effect of tDCS. (**A**) For visualization purposes, axial slices of the mean probabilistic tracts between L-DLPFC (in blue) and left thalamus (in green) are shown. This mean probabilistic tract was used as a mask to derive individual’s FA within the L-DLPFC–thalamic tract. (**B**) There was a significant positive correlation between the mean FA within the L-DLPFC–thalamic tract and pain intensity decrease after anodal tDCS (Anodal_[Pre-stimulation−Stimulation]_; r = 0.654, p < 0.01) but not during sham (Sham_[Pre-stimulation−Stimulation]_; r = 0.300, p = 0.23). No data points were found to be outliers using Grubbs’ test (p < 0.01).

**Figure 5:**
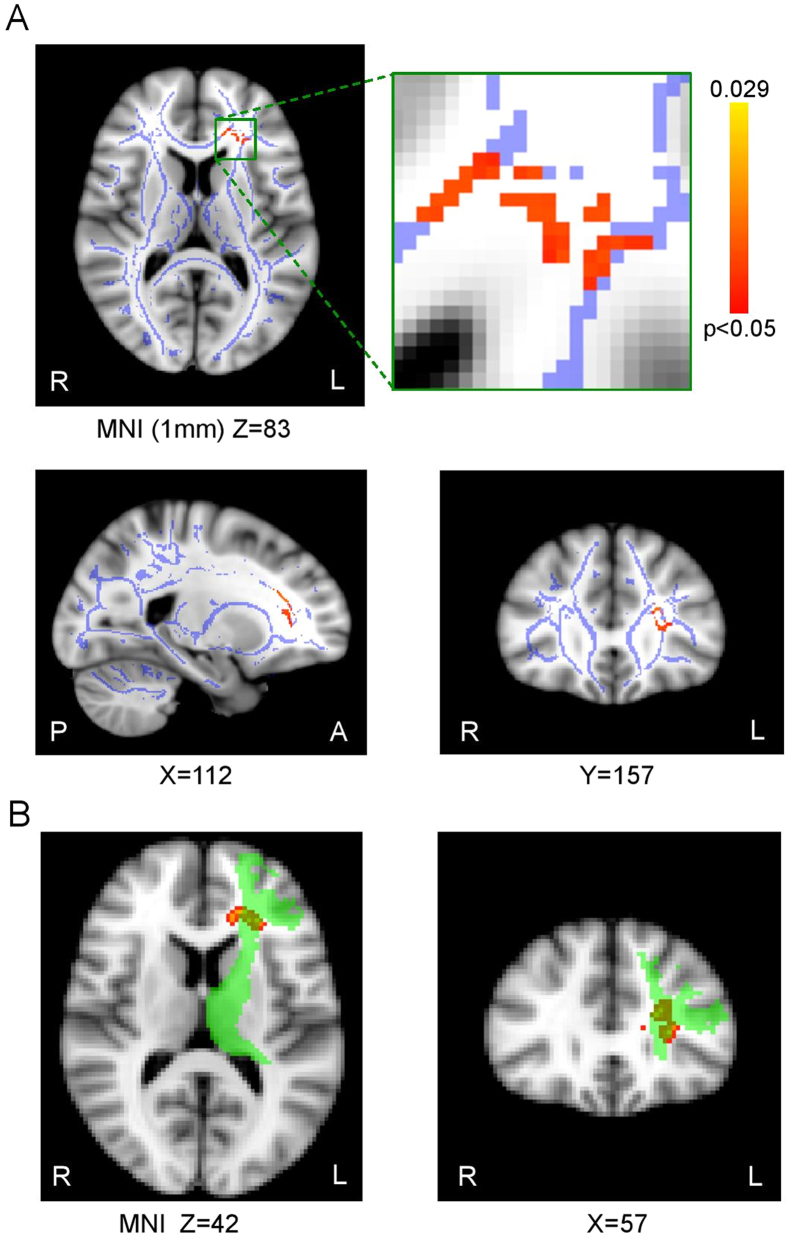
Structural connectivity correlating tDCS-induced analgesia is specific to L-DLPFC-thalamic pathway (FWE-corrected p < 0.05). (**A**) TBSS analysis describing the relationship between white matter integrity and tDCS induced pain intensity changes (i.e. Sham_[Stimulation−Pre-stimulation]_ − Anodal_[Stimulation−Pre-stimulation]_) across the whole white matter FA skeleton (in blue) (p < 0.05, FWE-corrected). (**B**) The only regions in which there was a significant correlation (in red-yellow) anatomically corresponded to the L-DLPFC-thalamic pathway identified using probabilistic tractography (in green; from Fig. 4A).

**Figure 6:**
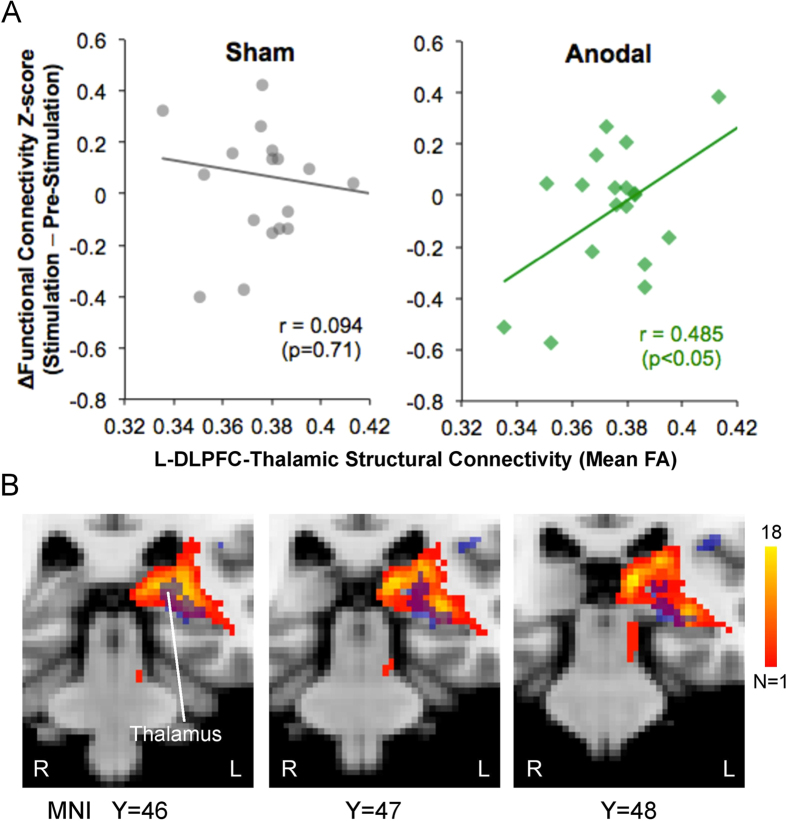
L-DLPFC-thalamic structural integrity correlates with functional connectivity during tDCS. (**A**) The relationship between structural and functional connectivity of L-DLPFC and thalamus during tDCS was investigated. The correlation between the two measures was significant for the anodal tDCS session (r = 0.485, p < 0.05) but not for the sham tDCS session (r = −0.094, p = 0.71). No data points were found to be outliers using Grubbs’ test (p < 0.01). (**B**) Activated voxels (in blue) from perfusion map between anodal and sham tDCS sessions (Sham_[Stimulation−Pre-stimulation]_ − Anodal_[Stimulation−Pre-stimulation]_; Fig. 3A) were overlaid on the white matter pathways identified using probabilistic tractography (in yellow-red). An overlap could be observed between the thresholded tracts and left thalamic voxels with rCBF changes.

**Figure 7:**
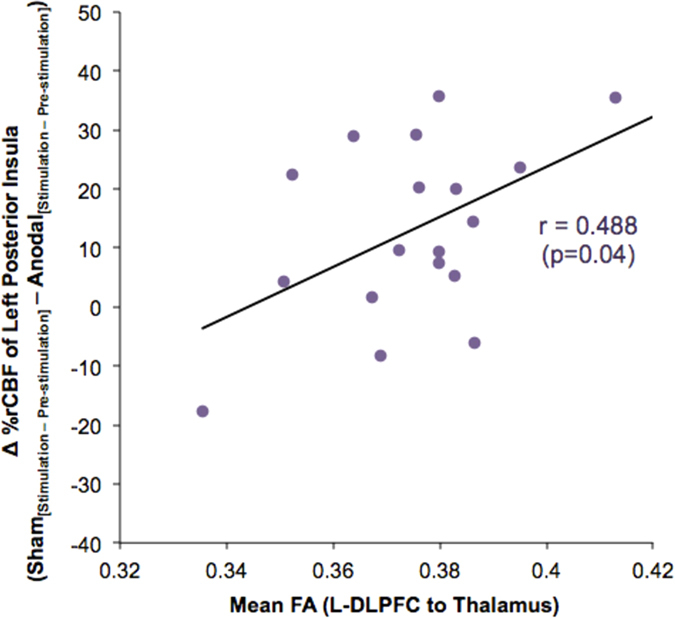
L-DLPFC-thalamic structural integrity correlates with changes in posterior insula rCBF. The relationship between structural connectivity of L-DLPFC-thalamic pathway and rCBF changes in the activated voxels (as shown in Fig. 3A) of posterior insula (Sham_[Stimulation−Pre-stimulation]_ − Anodal_[Stimulation−Pre-stimulation]_) was investigated. The correlation between the two measures was significant (r = 0.488, p = 0.04). No data points were found to be outliers using Grubbs’ test (p < 0.01).

The second-level analysis used paired t-tests to perform intra-subject comparisons between scans. These individual analyses were then submitted to a third and final level of analysis for the averaged group comparison of rCBF across periods (mixed effect; Z > 2.0, cluster corrected for multiple comparisons at p < 0.01). This sequence of analyses allowed for consideration of both the intra-scan and intra-subject (anodal and sham sessions) variances, thus minimizing the bias from any single session or participant. For visualization of the perfusion time courses of left posterior insula (Fig. 3B), extreme outliers (Grubbs’ test across subjects; p < 10^−10^) were removed to improve clarity.

#### Functional Connectivity Measures

We used a normalized Pearson correlation coefficient as a measure of functional connectivity between L-DLPFC and left thalamus, a method used in previous literature^3^. The mean perfusion-subtracted time courses of the two regions were extracted for the pre-stimulation and stimulation periods separately. Their respective Pearson correlation coefficient was then calculated for each period and normalized to Z-score with Fisher transformation. We obtained our functional connectivity measure by computing the difference within subjects between these two correlation Z-scores. Finally, this was covaried with individual FA within the L-DLPFC-thalamic tract across subjects.

### Anatomical regions of interest

The L-DLPFC mask covered Brodmann areas 8 A, 46, and 9/46, which have similar cytoarchitectonic features with a well-developed layer 4 67. The demarcation is limited posteriorly by the superior precentral sulcus on the lateral surface^68^. This extends anteriorly until we could no longer define the middle frontal gyrus at the border of the frontopolar area 10. In addition, the superior border was identified as the superior frontal sulcus. Any voxels below 50% probability of being gray matter, as determined by the MNI152 gray matter probabilistic mask, were excluded. Left M1 mask was drawn anatomically in MNI space and included the anterior portion of the left central sulcus and the posterior portion of the left precentral gyrus^60^. It extends from the dorsal border of the lateral ventricles to the dorsal surface and from the interhemispheric fissure to the left lateral surface. The thalamus mask was derived from the Harvard-Oxford probabilistic atlas at a high 75% threshold to prevent the inclusion of any nearby basal ganglia structures. For the mask below the electrode, we manually drew a 2 × 2 × 1 cm^3^ area underneath the center of gravity (COG) of the active electrode and directly superficial to the COG of the L-DLPFC region for our ROI analysis. All ROI masks were manually examined to ensure their validity. Other gray and white matter structures were identified with Harvard-Oxford probabilistic atlas, Johns Hopkins University White-Matter Tractography Atlas, or Juelich Histological Atlas.

### Data Analysis – Diffusion imaging

#### Diffusion Imaging Preprocessing

All data analyses were performed with tools from FSL version 4.1.7 66. Eddy current correction was initially used on the raw DTI images to remove motion artefacts and distortions associated with the applied gradient directions.

#### Probabilistic Tractography

In this study, the *a priori* hypothesis was that the pain modulatory effects of tDCS would relate to the strength of the structural connectivity between L-DLPFC and left thalamus. We fitted a multi-fiber diffusion model^30^ that estimated probability distributions on the fiber directions at each brain voxel in the diffusion space of each subject. To perform probabilistic tractography in the standard space for each of the 18 subjects, we fed both the warpfields generated during the first steps of TBSS (see below) and their corresponding inversed warpfields into the tractography algorithm. Tractography was carried out in standard space between every voxel of the L-DLPFC ROI and the thalamus ROI for all subjects. We created an exclusion region in the standard space at the sagittal midline to reject spurious inter-hemispheric fibers that cross the corpus callosum.

For each tractography, we generated 5,000 samples from each voxel of the two ROI masks to build up a connectivity distribution. Only those that passed through both masks and none of the regions in the exclusion mask were retained. We then thresholded individually the reconstructed white matter pathways at 2% of the total number of generated tracts in each subject. The individual FA, which served in this study as a quantitative measure of the structural connectivity, was extracted and averaged in each corresponding thresholded tract.

#### Tract-based Spatial Statistics (TBSS)

TBSS (version 1.2), an FSL voxelwise method for performing analysis of white matter microstructure without an *a priori* hypothesis^31^,^66^, was performed on individual FA maps generated from a tensor-model fit. While voxel-based approaches are based purely on non-linear registration, TBSS increases the sensitivity and the interpretability of the results because it projects the nearest maximum FA values onto a skeleton derived from the mean FA image. This additional projection step can remove the effect of cross-subject spatial variability that remains after the non-linear registration.

In this study, TBSS analysis, as it was exploring the whole white matter skeleton without *a priori* assumption, was used to verify the specificity of our results obtained in the L-DLPFC-thalamic pathways based on prior hypothesis. Regression analyses were carried out with permutation-based nonparametric tests (5,000 permutations). Results were considered significant at p < 0.05, fully corrected for multiple comparisons using threshold-free cluster enhancement (TFCE).

### Statistics

Individual statistical tests of structural connectivity, functional connectivity, and pain intensity ratings were performed with SPSS software (version 18.0, IBM, Armonk, NY, USA) or Microsoft Excel (version 14.4.4, Microsoft, Redmond, WA, USA). Unless otherwise specified, paired t-tests (two-tailed) were used to determine statistical significance. Results with p < 0.05 were considered significant.

## Additional Information

**How to cite this article**: Lin, R. L. *et al*. Structural Connectivity Variances Underlie Functional and Behavioral Changes During Pain Relief Induced by Neuromodulation. *Sci. Rep.* **7**, 41603; doi: 10.1038/srep41603 (2017).

**Publisher's note:** Springer Nature remains neutral with regard to jurisdictional claims in published maps and institutional affiliations.

## Supplementary Material

Supplementary Information
