# Supplementary material for: Structural Connectivity Variances Underlie Functional and Behavioral Changes During Pain Relief Induced by Neuromodulation
Source: Sci Rep. 2017 Feb 2;7:41603. doi: 10.1038/srep41603 (PMC5288647; doi:10.1038/srep41603)
Supplement: Supplementary Information [file srep41603-s1.doc]

**SUPPLEMENTARY INFORMATION**

**Structural Connectivity Variances Underlie Functional and Behavioral Changes During Pain Relief Induced by Neuromodulation**

*Running Title:* Structure-Function Association and Pain Modulation

*Keywords*: pain, neuromodulation, transcranial direct current stimulation, arterial spin labelling, diffusion imaging, analgesia

Richard L. Lin^1,2,*^, Gwenaëlle Douaud^1^, Nicola Filippini^1,3^, Thomas W. Okell^1^, Charlotte J. Stagg^1,4^, Irene Tracey^1,2^

^1^Oxford Centre for Functional Magnetic Resonance Imaging of the Brain (FMRIB Centre) &

^2^Nuffield Division of Anaesthetics, Nuffield Department of Clinical Neurosciences &

^3^Nuffield Department of Clinical Neurosciences &

^4^Oxford Centre for Human Brain Activity, Department of Psychiatry, University of Oxford, Oxford OX1 3UH, United Kingdom

*Correspondence: [rllin@mit.edu](mailto:rllin@mit.edu )

**Supplementary Data**

**Supplementary Figure 1**. **Volunteer response to capsaicin.** Mean pain intensity ratings of the eighteen subjects with topical application of 1% capsaicin cream on the right calf in the screening session. In the 30-minute interval after subjects reached a rating of 5 on a 10-point continuous rating scale, the pain scores largely stabilized between scores of 5 and 7. The continuous pain intensity scale was anchored between the extremes of “None” and “Worst Imaginable.”

**Supplementary Figure 2**. **Questionnaire results on electrical stimulation sensations after anodal and sham tDCS sessions**. No significant differences were observed in all surveyed categories, which consisted of tingling (p=0.83), itching (p=0.47), burning (p=0.79), tiredness (p=0.39), nervousness (p=0.67), concentration (p=0.71), headache (p=0.55), visual problems (p=0.33), and visible flash (p=0.33). Presence of visual flash was determined with a binary yes/no question, while the others were rated from 1 to 5 (1=mild, 2=moderate, 3=average, 4=severe, and 5=intolerable). If subjects did not experience a particular sensation during stimulation, their rating in that category was marked 0. These results suggested that successful blinding was achieved with sham tDCS. All statistical tests were performed with two-tailed t-tests. Error bars denote the standard error of means across subjects.

**Supplementary Figure 3**. **Perfusion activity map of gray matter associated with Anodal_[Stimulation – Pre-stimulation]_** (mixed effects; Z>2, FWE-corrected at p<0.01). L-DLPFC (denoted by the yellow mask), M1, SI, and the visual cortex showed increased rCBF during stimulation from pre-stimulation period. (DLPFC = dorsolateral prefrontal cortex; M1 = primary motor cortex; SI = primary somatosensory cortex)

**Supplementary Figure 4**. **Perfusion activity map of gray matter associated with Sham_[Stimulation – Pre-stimulation]_** (mixed effects; Z>2, FWE-corrected at p<0.01). SI and visual cortex were associated with increased rCBF during stimulation from the pre-stimulation period, while decreases were observed in the right middle temporal gyrus. (SI = primary somatosensory cortex)

**Supplementary Figure 5**. **Structural connectivity between L-M1 and L-DLPFC, left thalamus, or left posterior insula does not correlate with analgesic effect of tDCS.** As we observed tDCS effects on the M1 (Fig. 3), an analysis exploring whether there were relationships between tDCS induced analgesia and the structural integrity of M1 with key pain-related regions was performed. No significant correlations were found between pain intensity decrease after anodal (Anodal[Pre-stimulation – Stimulation]) or sham tDCS (Sham[Pre-stimulation – Stimulation]) and the structural integrity of (A) L-M1-DLPFC, (B) L-M1-thalamus, and (B) L-M1-poserior insula.

**Supplementary Text**

In this study, we found a behavioral and functional dependence on structural connectivity during pain neuromodulation. Specifically, the strength of the L-DLPFC–thalamic connection correlated with the magnitude of pain relief and the regions' functional inter-correlation. These findings underline the importance of the interrelationship between structural connection and functional and behavioral measures in neuroscience. We provide five supplementary figures to support our argument.

To demonstrate the efficacy of capsaicin in inducing ongoing pain, we reported the pain intensity ratings of the eighteen capsaicin responders (and excluded the one subject who withdrew) following topical application of capsaicin cream (Supplementary Fig. 1). The figure shows the average ratings after the subjects reached a score of 5 on a continuous rating scale from 0 (“None”) to 10 (“Worst Imaginable”). The pain intensity ratings of all subjects were within 5 and 7 on the scale. Besides a small incline at the beginning and decline at the end, the mean pain intensity rating remained steady throughout the 30-minute interval.

After each tDCS session, subjects were given an exit questionnaire to determine whether they experienced different sensations between anodal and sham tDCS. No significant differences were found with paired t-tests in the questions, which included tingling (p=0.83), itching (p=0.47), burning (p=0.79), tiredness (p=0.39), nervousness (p=0.67), concentration (p=0.71), headache (p=0.55), visual problems (p=0.33), and visible flash (p=0.33) (Supplementary Fig. 2). This suggests that sham tDCS was not physically discernible from anodal tDCS for the subjects.

Collected across the cohort of 18 volunteers, perfusion fMRI data was then used to generate the perfusion maps to infer changes in gray matter activity across the whole brain. During pain modulation by anodal L-DLPFC tDCS, there was an increased rCBF in L-DLPFC, M1, SI, and the visual cortex compared to the pre-stimulation period (Supplementary Fig. 3). No significant rCBF decreases were observed, and the effect on the frontal cortex was lateralized to the stimulated hemisphere (left). In comparison, neuromodulation by sham tDCS was associated with fewer regions of significant rCBF changes (Supplementary Fig. 4). Increases were observed in the visual cortex and SI from the pre-stimulation period, while decreases were associated with the right middle temporal gyrus. No significant changes were observed in the L-DLPFC.

As we observed tDCS effects on the M1 (Fig. 3), we sought to determine whether M1 activity may have led to subjects’ behavioral changes with anodal tDCS. We investigated the relationships between the structural integrity of L-M1-DLPFC, L-M1-posterior insula, or L-M1-thalamus and tDCS induced analgesia and found no significant correlations (Supplementary Fig. 5).

Areas that show functional change between stimulation conditions (anodal, sham, or none) are shown in Supplementary Tables 1-3.
